# Supplementary material for: Knowledge, attitude and practice of cervical cancer screening among women infected with HIV in Africa: Systematic review and meta-analysis
Source: PLoS One. 2021 Apr 8;16(4):e0249960. doi: 10.1371/journal.pone.0249960 (PMC8031808; doi:10.1371/journal.pone.0249960)
Supplement: S1 Appendix — (PDF) [file pone.0249960.s001.pdf]

Knowledge, attitude and practice of cervical cancer screening among women infected with HIV in Africa: Systematic review and meta-analysis

Agajie Bogale, Tilahun Habtemariam, Jemal Haidar Ali, Getnet Mitikie Kassie

To enable PROSPERO to focus on COVID-19 registrations during the 2020 pandemic, this registration record was automatically published exactly as submitted. The PROSPERO team has not checked eligibility.

Citation

Agajie Bogale, Tilahun Habtemariam, Jemal Haidar Ali, Getnet Mitikie Kassie. Knowledge, attitude and practice of cervical cancer screening among women infected with HIV in Africa: Systematic review and meta-analysis. PROSPERO 2020 CRD42020210879 Available from: https://www.crd.york.ac.uk/prospero/display_record.php?ID=CRD42020210879

Review question

What is the knowledge, attitude and practice of women infected with HIV towards cervical cancer screening in Africa?

Searches

We will use PubMed/MEDLINE, Scopus, ScienceDirect, Web of Science, Cumulative Index of Nursing and allied Health Sciences (CINAHL) and Google Scholar electronic biomedical databases for extraction of data for English language articles published within ten years period till last August 2020.

Search strategy

The search mainly focuses on knowledge, attitude and practice score towards cervical cancer screening among women infected with HIV in Africa. During searching, the following keywords (terms) were used: knowledge, attitude, practice, cervical cancer, uterine cervical neoplasms, cervical cancer screening, human immunodeficiency virus or HIV, infect*, positive, women, female*, Africa.

Types of study to be included

Observational study designs, mainly cross-sectional type and cohort study.

Condition or domain being studied

This review depicted that there was variability on the knowledge score, attitude and practice of cervical cancer and prevention aspects among women participants in different countries. Moreover, the study among vulnerable population meaning those women living with HIV were very limited. This is, therefore, this review was aimed to figure out the knowledge score, attitude and practice of cervical cancer and prevention aspects among HIV infected women in the continent of Africa in which HIV prevalence was high compared with developed countries.

Participants/population

HIV infected women

Intervention(s), exposure(s)

The inclusion was restricted to original research articles published in English language. Those studies that did not clearly state the outcome measures (i.e. knowledge, attitude and practice towards cervical cancer screening), study population different from HIV infected women or females, duplication citations, review articles were excluded in this review.

Comparator(s)/control

No comparator in this review.

Main outcome(s)

We will estimate the pooled knowledge of HIV infected women on cervical cancer screening with its 95% Confidence Interval (CI) using random effects meta-analysis model assuming the true effect size varies

between studies.

* Measures of effect

We will use odds ratio for the effect measure of the main outcome in this review.

Additional outcome(s)

Attitude and practice of HIV infected women towards cervical cancer screening.

* Measures of effect

We will use odds ratio for the effect measure of additional outcomes in this review.

Data extraction (selection and coding)

Eligible abstract and/or full text of the articles extracted by considering the outcome variables and the characteristics of participants such as age range, mean or median age, sex, HIV sero-status, were extracted information for the study. And also, the study characteristics such as first author, year of publication, study setting, study location or country, study design, sample size, knowledge score, attitude and practice were extracted for the study.

Risk of bias (quality) assessment

The quality of selected studies was assessed using Quality Assessment Tool for Observational Cohort and Cross-Sectional Studies - NHLBI, NIH which contains 14 items. This assessment tool mainly focused on research question, study population, eligibility criteria (inclusion and exclusion criteria of study participants), sample size justification, exposure measures and assessment, sufficient time frame to see an effect, outcome measures and blinding of outcome assessors, follow up rate, and statistical analysis. The quality assessment is rated as good, fair and poor. The maximum score indicating high quality was 14, with the lowest possible score being zero

Strategy for data synthesis

We will use STATA version 14 (using metaprop command and by entering all the required variables for analysis). The model will be random effects meta-analysis model assuming the true effect size varies between studies. Data were presented in forest plot. Heterogeneity in the prevalence of different studies assessed using heterogeneity $?^2$ (x2) with a degree of freedom. The P value obtained by comparing the statistic with a x2 distribution with K-1 degree of freedom (where K is the number of studies). The quantity, which we call I^2 describes the percentage of total variation across studies that is due to heterogeneity rather than chance and it is represented as low (25%), moderate (50%) and high (75%) values.

Analysis of subgroups or subsets

We can use meta-regression model to rule out heterogeneity for sample size and years of publication.

Contact details for further information

Agajie Likie Bogale
agalb.2000@yahoo.com

Organisational affiliation of the review

Ethiopian Public Health Institute, Addis Ababa University

Review team members and their organisational affiliations

Mr Agajie Bogale. Ethiopian Public Health Institute, Addis Ababa University
Professor Tilahun Habtemariam. Addis Ababa University
Professor Jemal Haidar Ali. Addis Ababa University
Professor Getnet Mitikie Kassie. Addis Ababa University

Type and method of review

Meta-analysis, Systematic review

Anticipated or actual start date

01 June 2020

Anticipated completion date

30 November 2020

Funding sources/sponsors
There is no need of funding for this review.

Conflicts of interest

Language
English

Country
Ethiopia

Stage of review
Review Ongoing

Subject index terms status
Subject indexing assigned by CRD

Subject index terms
MeSH headings have not been applied to this record

Date of registration in PROSPERO
24 October 2020

Date of first submission
23 September 2020

Stage of review at time of this submission

Stage	Started	Completed
Preliminary searches	Yes	Yes
Piloting of the study selection process	Yes	Yes
Formal screening of search results against eligibility criteria	Yes	Yes
Data extraction	No	No
Risk of bias (quality) assessment	No	No
Data analysis	No	No

The record owner confirms that the information they have supplied for this submission is accurate and complete and they understand that deliberate provision of inaccurate information or omission of data may be construed as scientific misconduct.

The record owner confirms that they will update the status of the review when it is completed and will add publication details in due course.

Versions
24 October 2020

This information has been provided by the named contact for this review. CRD has accepted this information in good faith and registered the review in PROSPERO. The registrant confirms that the information supplied for this submission is accurate and complete. CRD bears no responsibility or liability for the content of this registration record, any associated files or external websites.
