# Supplementary material for: Knowledge, attitude and practice of cervical cancer screening among women infected with HIV in Africa: Systematic review and meta-analysis
Source: PLoS One. 2021 Apr 8;16(4):e0249960. doi: 10.1371/journal.pone.0249960 (PMC8031808; doi:10.1371/journal.pone.0249960)
Supplement: S1 Table — (DOC) [file pone.0249960.s002.doc]

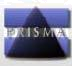
PRISMA 2009 Checklist


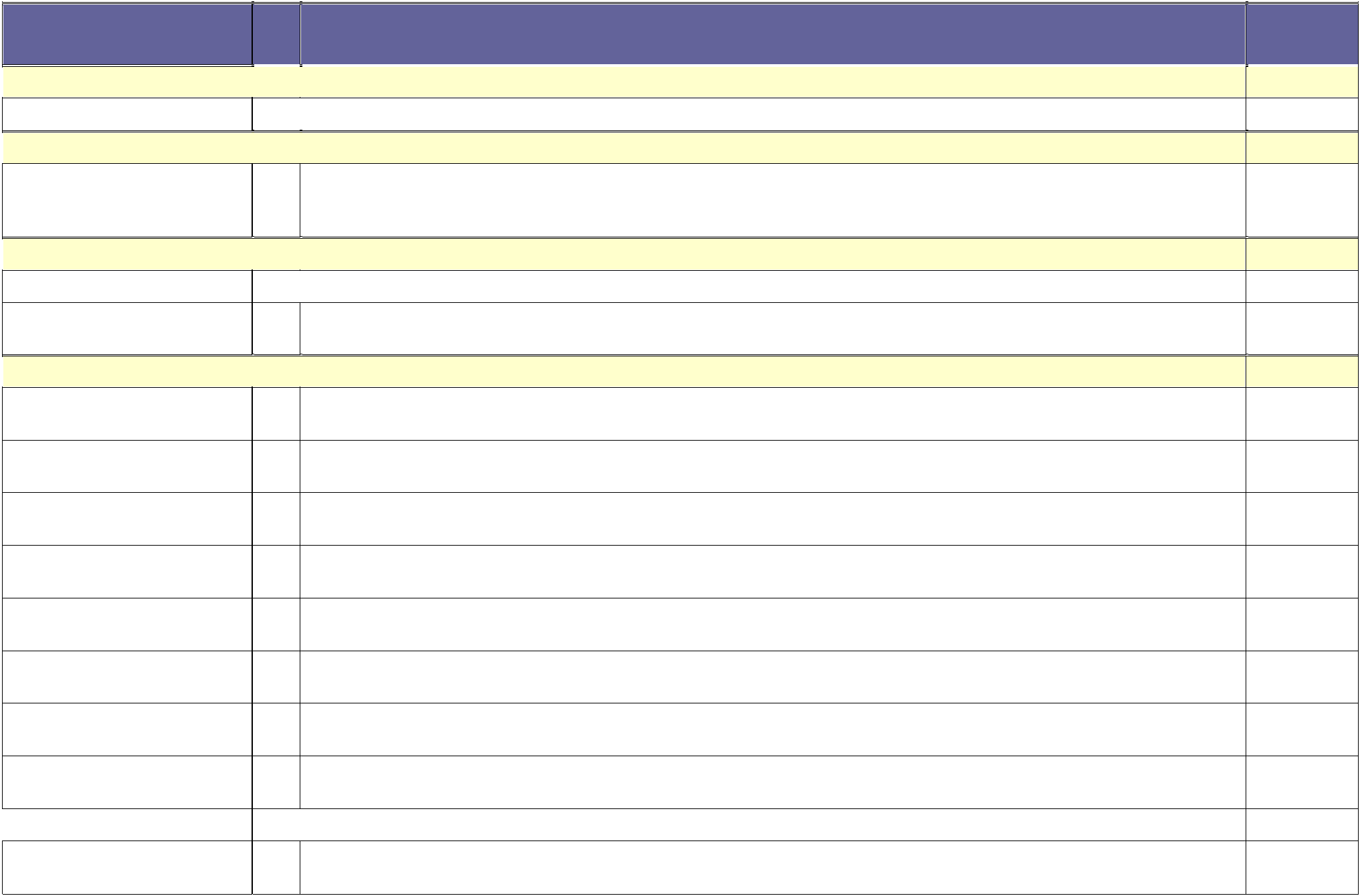


**Section/topic**


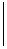
 **TITLE**

Title


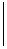
 **ABSTRACT**

Structured summary


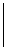
 **INTRODUCTION**

Rationale

Objectives


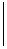
 **METHODS**

Protocol and registration

Eligibility criteria

Information sources

Search

Study selection

Data collection process

Data items

Risk of bias in individual studies


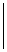
 Summary measures Synthesis of results

| **# Checklist item** | | |  | **Reported** |
| --- | --- | --- | --- | --- |
|  |  |  |  | **on page #** |
|  |  |  |  |  |
|  |  |  |  |  |

1
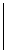
 Identify the report as a systematic review, meta-analysis, or both. "Reported on Page #1"

1. Provide a structured summary including, as applicable: background; objectives; data sources; study eligibility criteria, participants, and interventions; study appraisal and synthesis methods; results; limitations; conclusions and implications of key findings; systematic review registration number. "Reported on Page # 2"

3
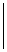
 Describe the rationale for the review in the context of what is already known. "Reported on Page # 4"

1. Provide an explicit statement of questions being addressed with reference to participants, interventions, comparisons, outcomes, and study design (PICOS). "Reported on Page # 4"
2. Indicate if a review protocol exists, if and where it can be accessed (e.g., Web address), and, if available, provide registration information including registration number. "Registered at PROSPERO; CRD42020210879"
3. Specify study characteristics (e.g., PICOS, length of follow-up) and report characteristics (e.g., years considered, language, publication status) used as criteria for eligibility, giving rationale. "Reported on Page # 5"
4. Describe all information sources (e.g., databases with dates of coverage, contact with study authors to identify additional studies) in the search and date last searched. "Reported on Page # 4 & 5"
5. Present full electronic search strategy for at least one database, including any limits used, such that it could be repeated. "Reported on Page # 5"
6. State the process for selecting studies (i.e., screening, eligibility, included in systematic review, and, if applicable, included in the meta-analysis). "Reported on Page # 6"
7. Describe method of data extraction from reports (e.g., piloted forms, independently, in duplicate) and any processes for obtaining and confirming data from investigators. "Reported on Page # 7"
8. List and define all variables for which data were sought (e.g., PICOS, funding sources) and any assumptions and simplifications made. "Table 1, and funding sources reported at Page 15"
9. Describe methods used for assessing risk of bias of individual studies (including specification of whether this was done at the study or outcome level), and how this information is to be used in any data synthesis. "Reported on Page # 7".

13
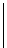
 State the principal summary measures (e.g., risk ratio, difference in means). "Reported on Page # 9 &10"

1. Describe the methods of handling data and combining results of studies, if done, including measures of consistency (e.g., I^2^) for each meta-analysis. "Reported on Page # 8"

Page 1 of 2


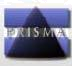
PRISMA 2009 Checklist


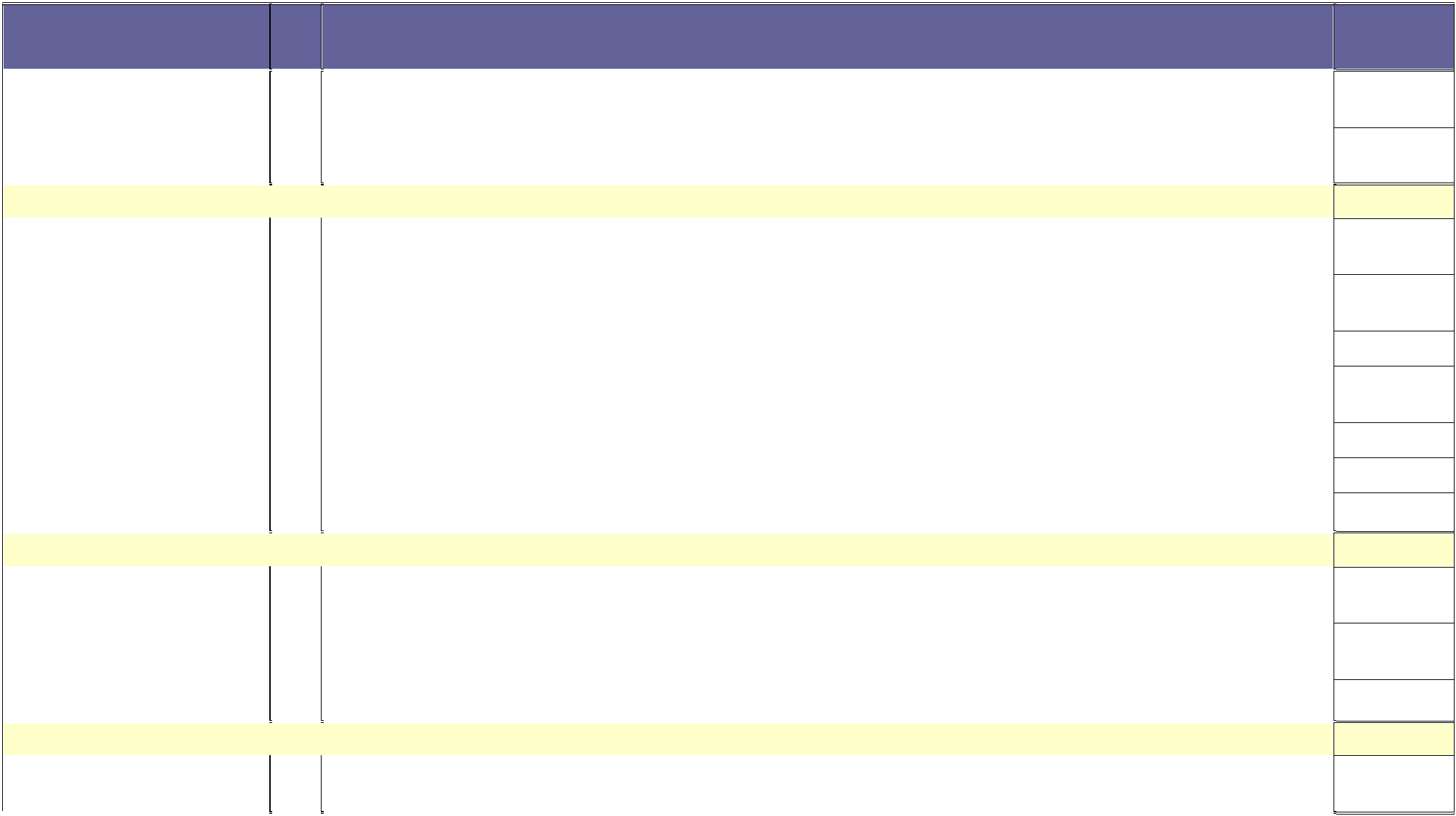


| **Section/topic** | **#** | |  | **Checklist item** |
| --- | --- | --- | --- | --- |
|  |  |  |  |  |
| Risk of bias across studies | 15 | |  | Specify any assessment of risk of bias that may affect the cumulative evidence (e.g., publication bias, selective |
|  |  |  |  | reporting within studies). "Reported on Page # 8" |
|  |  | |  |  |
| Additional analyses | 16 | |  | Describe methods of additional analyses (e.g., sensitivity or subgroup analyses, meta-regression), if done, indicating |
|  |  |  |  | which were pre-specified. "Reported on Page # 10" |
|  |  |  |  |  |
| **RESULTS** |  |  |  |  |
| Study selection | 17 | |  | Give numbers of studies screened, assessed for eligibility, and included in the review, with reasons for exclusions at |
|  |  |  |  | each stage, ideally with a flow diagram. "Reported on Page # 6" |
|  |  | |  |  |
| Study characteristics | 18 | |  | For each study, present characteristics for which data were extracted (e.g., study size, PICOS, follow-up period) and |
|  |  |  |  | provide the citations. "Reported on Page # 8, Table 1" |
|  |  | |  |  |
| Risk of bias within studies | 19 | |  | Present data on risk of bias of each study and, if available, any outcome level assessment (see item 12).  "Reported on Page # 7, Table 1" |
|  |  | |  |  |
| Results of individual studies | 20 | |  | For all outcomes considered (benefits or harms), present, for each study: (a) simple summary data for each |
|  |  |  |  | intervention group (b) effect estimates and confidence intervals, ideally with a forest plot. "Reported on Page # 9, Fig 2-4" |
|  |  | |  |  |
| Synthesis of results | 21 | |  | Present results of each meta-analysis done, including confidence intervals and measures of consistency.  "Reported on Page # 10, Table 2" and "Fig 2-5". |
|  |  | |  |  |
| Risk of bias across studies | 22 | |  | Present results of any assessment of risk of bias across studies (see Item 15). "Reported on Page # 8" and Fig.5a-c" |
|  |  | |  |  |
| Additional analysis | 23 | |  | Give results of additional analyses, if done (e.g., sensitivity or subgroup analyses, meta-regression [see Item 16]).  "Reported on Page # 10" |
|  |  |  |  |  |
| **DISCUSSION** |  |  |  |  |
| Summary of evidence | 24 | |  | Summarize the main findings including the strength of evidence for each main outcome; consider their relevance to |
|  |  |  |  | key groups (e.g., healthcare providers, users, and policy makers). "Reported on Page # 11" |
|  |  | |  |  |
| Limitations | 25 | |  | Discuss limitations at study and outcome level (e.g., risk of bias), and at review-level (e.g., incomplete retrieval of |
|  |  |  |  | identified research, reporting bias). "Reported on Page # 13" |
|  |  | |  |  |
| Conclusions | 26 | |  | Provide a general interpretation of the results in the context of other evidence, and implications for future research.  "Reported on Page # 14" |
|  |  |  |  |  |
| **FUNDING** |  |  |  |  |
| Funding | 27 | |  | Describe sources of funding for the systematic review and other support (e.g., supply of data); role of funders for the |
|  |  |  |  | systematic review. "Reported on Page # 15" |
|  |  |  |  |  |

**Reported on page #**

*From:* Moher D, Liberati A, Tetzlaff J, Altman DG, The PRISMA Group (2009). Preferred Reporting Items for Systematic Reviews and Meta-Analyses: The PRISMA Statement. PLoS Med 6(7): e1000097. doi:10.1371/journal.pmed1000097

For more information, visit: **www.prisma-statement.org**.
