# Supplementary material for: Knowledge, attitude and practice of cervical cancer screening among women infected with HIV in Africa: Systematic review and meta-analysis
Source: PLoS One. 2021 Apr 8;16(4):e0249960. doi: 10.1371/journal.pone.0249960 (PMC8031808; doi:10.1371/journal.pone.0249960)
Supplement: S2 Table — (DOCX) [file pone.0249960.s003.docx]

**S2 Table. The results of the individual components of the quality assessment**

| **First author** | **Year** | **Individual component score** | **Percentage score** | **Remark** |
| --- | --- | --- | --- | --- |
| Solomon et al[24] | 2019 | 8/8 | 100% |  |
| Shiferaw et al[25] | 2018 | 8/8 | 100% |  |
| Mitchell et al[26] | 2017 | 7/8 | 87.5% |  |
| Stuart et al[27] | 2019 | 5/8 | 62.5% |  |
| Adibe&Aluh[28] | 2017 | 6/8 | 75.0% |  |
| Belglaiaa et al[29] | 2018 | 5/8 | 62.5% |  |
| Rosser et al[30] | 2015 | 7/8 | 87.5% |  |
| Maree &Moitse[31] | 2014 | 5/8 | 62.5% |  |

**Note**: The quality assessment tool used for observational cohort and cross-sectional studies of 14 items, while almost all included studies are cross-sectional type which address 8 items from the total 14 items. The remaining 6 fit for cohort studies which is not applicable for the cross-sectional type.
