# Supplementary material for: Knowledge, attitude and practice of cervical cancer screening among women infected with HIV in Africa: Systematic review and meta-analysis
Source: PLoS One. 2021 Apr 8;16(4):e0249960. doi: 10.1371/journal.pone.0249960 (PMC8031808; doi:10.1371/journal.pone.0249960)
Supplement: Attachment — Submitted filename: Response to Reviewers-PONE.docx [file pone.0249960.s004.docx]

Manuscript PONE-D-21-03081

Response to Editor and Reviewers

Thank you for the valued comments, and the effort made to improve our manuscript titled, Knowledge, attitude and practice of cervical cancer screening among women infected with HIV in Africa: Systematic review and meta-analysis. We are very grateful for the insightful comments and the input made. Based on the valuable suggestions forwarded, we revised the MS and incorporated all the comments as appropriate and highlighted the changes made.

Regarding the suggestion on grammatical errors, senior instructors and co-authors critically reviewed, and online Spell checker – grammar and spell check in English - Reverso was used to check the spelling and grammar of the manuscript submitted. As commented by all reviewers, the entire manuscript is reassessed and minor corrections are made accordingly in the revised version. It is a privilege to work with you in the future prospects considering quality of the review and hard work.

The followings are the line by line responses made for the valued suggestions and comments raised by your esteemed Editors and reviewers;

**Editor comments:**

**Comment 1. Check PLOS ONE's style requirements**

- Authors' response: Thank you for pointing out this issue. The revised manuscript followed PLOS ONE manuscript formatting guidelines, including use of level 1 heading for all major sections, bold type, 18pt font.

**Comment 2. Confirm that you have included all items recommended in the PRISMA checklist**

- Authors' response: Thank you. We confirmed that all the items in the PRISMA checklist are included and the search strategy is well described on page 5 of the manuscript.
- We tried to restrict the studies done within the last 10 years since we were interested to assess the recent/current knowledge, attitude and practice of HIV positive women towards cervical cancer screening.
- The results of the individual components of quality assessment are included as a supplemental file (S2 Table) as suggested.

**Comment 3. Include a table as part of main manuscript.**

- Authors' response: Thank you and we have inserted the tables as suggested in the revised version of the manuscript [page 8]

**Comment 4. State the financial disclosure**

- Authors' response. Comment incorporated [Page 16].

**Comment 5. PLOS requires an ORCID iD for the corresponding author**

- Author response. Thank you. Comment incorporated and the ORCID iD for the corresponding author's is 0000-0002-2794-8251

**Comment 6. Your ethics statement should only appear in the Methods section of your manuscript**

- Authors' response: Thank and the comments is incorporated. [Page 9]

**Comment 7. Include captions for your Supporting Information files at the end of your manuscript**

- Authors' response: Comment incorporated.

**Reviewers' Comments:**

| **Reviewers'** | **Comments** | **Authors response** | **Remark** |
| --- | --- | --- | --- |
| Reviewer #1 | Grammatical errors | Done as suggested. |  |
|  | Definite time frame for search | The search is already filtered by selecting 10 years in the left corner of database search till the time of data extraction which is August 2020. |  |
|  | The number of articles identified do not add-up to 131 | Thank you for pointing this out. The records identified via Google scholar (n=46) are covered due to font size and now visible. |  |
| Reviewer #2 | -I will worry about disparity in healthcare access creating a bias on KAP  - Most patients were from Ethiopia and the difference in health system from different countries can affect the generalizability of the result to whole of the African Continent  - Cervical cancer incidence correlate with a lower CD4 count | Thank you very much for your valuable comments and suggestions.  -Our review was conducted among HIV population from ART clinics with more or less similar characteristics in terms of awareness and practice.  - Only two articles are included in the analysis from Ethiopia, which fulfilled the eligibility criteria. [Table 1][Page 8]  - Sure! Immunosuppression hasan association with cervical cancer, but our aim here was to pool KAP estimates on cervical cancer among HIV population. |  |
| Reviewer #3 | -Define in the methodology what they meant by knowledge, attitude and practice (KAP) | Thank you for your valuable comments and suggestions.  - We included the operational definition of KAP under the methods section [Page 9].  - Other comments are also included in the revised manuscript.  - |  |
| Reviewer #4 | What are the specific parameters you used to decide whether KAP is prevalent or not? | Our aim in this regard is to pool the pocket studies which reported the proportions of KAP among HIV positive women. Based on this finding, our next step is to use structured questionnaire to address KAP issue in the subsequent original study and establish the average score.  Other comments also incorporated into the protocol. |  |
| Reviewer #5 | It will be informative to investigate the reason of such existing heterogeneity in knowledge, attitude and practice of cervical cancer screening among women infected with HIV in Africa. | Thank you very much for your valuable comment.  -We tried to use meta-regression to rule out heterogeneity on KAP considering variation in the sample size and years of publication might cause heterogeneity observed. [Page 11] and as you can see from Table 1, age, even reported in age range, mean age, etc., which is very difficult to run a meta-regression to rule-out heterogeneity due to age. |  |

Thank you very much,

Authors'
